# Exogenous Melatonin Enhances Salt-Stress Tolerance in *Festuca elata* via Growth and Physiological Improvements

**DOI:** 10.3390/plants14172661

**Published:** 2025-08-26

**Authors:** Bingqi Liu, Haimei Li, Xianhui Zhao, Junrui Wang, Yuting Zhang

**Affiliations:** College of Landscape Architecture and Forestry, Qingdao Agricultural University, Changcheng Road NO. 700, Qingdao 266109, China

**Keywords:** melatonin, salt stress, growth indicators, physiological ecology, *Festuca elata*

## Abstract

Salt stress is a major abiotic factor that inhibits plant growth. Melatonin (MT), an important plant growth regulator, can effectively enhance plant stress resistance. *Festuca elata*, a turfgrass species widely used in urban landscaping, was selected for this study to evaluate the regulatory effects of exogenous MT at different concentrations on its growth and development under salt stress. Indoor pot experiments were conducted using *Festuca elata* as the plant material. The experiment included a 250 mM NaCl salt-stress treatment and foliar application of five MT concentrations (0 μM, 50 μM, 150 μM, 250 μM, and 350 μM) to assess their effects under salt stress. The results showed that salt stress severely inhibited the growth of *Festuca elata*, while all tested MT concentrations significantly alleviated the damage. MT treatments improved leaf area and plant height and increased relative water content, soluble protein, proline, chlorophyll, and carotenoid contents. Additionally, MT reduced malondialdehyde accumulation and enhanced superoxide dismutase and peroxidase activities. Among the tested concentrations, 150 μM MT showed the most effective alleviation of salt stress, indicating its strong potential for promoting *Festuca elata* cultivation in saline environments.

## 1. Introduction

Salt stress is a major abiotic factor that inhibits plant growth [1]. Globally, soil salinization is steadily increasing [2,3]. In China, saline–alkali land covers approximately 1×10^8^ hm^2^. Particularly in northern China, low precipitation and high evaporation promote the accumulation of soluble salts in the topsoil [4,5]. Additionally, urban soil salinization is intensified by anthropogenic factors such as recycled water irrigation, regular fertilization, atmospheric salt deposition, and winter de-icing salt use in the northern regions. These practices degrade soil structure and significantly limit plant growth [6,7,8].

Salt stress imposes severe physiological challenges that impair plant growth and development [9,10,11]. First, high salt concentrations disrupt the ion homeostasis of plant cells, leading to excessive Na^+^ accumulation and K^+^ loss, which triggers ion toxicity effects [12]. Second, salt stress induces a burst of reactive oxygen species (ROS), causing membrane lipid peroxidation, protein denaturation, and DNA damage [13]. Additionally, salt stress interferes with the plant’s water balance, resulting in osmotic stress that induces stomatal closure, reduces photosynthetic rates, and inhibits the uptake of essential nutrients. These physiological disturbances ultimately manifest as growth retardation, developmental inhibition, reduced biomass, and even plant death [14]. Under 200 mM NaCl stress, the Na^+^ content in the leaves of all four turfgrass species increased significantly, while the relative water content (RWC) decreased markedly. Consequently, turf quality, leaf width, and leaf length were also significantly reduced [15]. Similarly, Jameel et al. [16] reported that 300 mM NaCl stress significantly reduced photosynthetic pigments while increasing malondialdehyde (MDA) and H_2_O_2_ contents, and significantly reduced root and stem length, leaf area, fresh weight, and dry weight in tomato plants, thereby inhibiting growth. Studies show that plants initiate physiological responses before visible morphological changes occur under salt stress, using strategies such as enhanced antioxidant activity and osmotic regulation to mitigate damage [17,18]. Plants enhance the activities of antioxidant enzymes, including superoxide dismutase (SOD) and peroxidase (POD), to scavenge reactive oxygen species (ROS). Concurrently, they accumulate osmoregulatory substances such as proline (Pro) and soluble protein (SP) to maintain cellular osmotic equilibrium. These adaptive responses collectively mitigate membrane lipid peroxidation damage, preserve ion homeostasis, and sustain normal plant growth and development [19]. However, prolonged high-salinity exposure leads to excessive Na^+^ and Cl^−^ accumulation, causing both osmotic and ionic stress. This results in overproduction of reactive ROS, collapse of antioxidant defenses, and oxidative damage to organelle membranes. Ultimately, this disrupts metabolic homeostasis, compromises cellular integrity, and severely inhibits plant growth [20,21].

Melatonin (N-acetyl-5-methoxytryptamine, MT) functions as an effective plant growth regulator and antioxidant that eliminates free radicals and plays a key role in mitigating salt stress in plants [22]. MT significantly enhances the stability of the photosynthetic system. Studies have demonstrated that under salt-stress conditions, plants treated with MT exhibit markedly higher contents of chlorophyll (Chl), and carotenoid (Car) compared to control groups [23]. Furthermore, MT mitigates salt stress by regulating osmotic balance. It not only directly promotes the accumulation of osmoregulatory substances such as Pro and soluble sugars but also enhances antioxidant enzyme activity and reduces MDA content, thereby effectively alleviating membrane system damage [24]. Zahedi et al. [25] demonstrated that foliar application of melatonin to salt-stressed olive seedlings significantly enhanced photosynthetic pigment content, osmoregulatory substance accumulation, and antioxidant enzyme activities in leaves, thereby effectively alleviating salt stress in the seedlings. Similarly, Liu et al. [26] found that foliar application of 25 µM and 50 µM MT significantly increased soluble sugar, proline, and chlorophyll contents while decreasing MDA levels in *Trifolium pretense*, enabling it to maintain normal growth under salt stress. Similar beneficial effects of melatonin application have been consistently observed across multiple crop species, including maize [27], rice [28], sweet potato [22], and cotton [29]. These reproducible findings strongly support that foliar melatonin spraying represents an effective strategy for alleviating salt stress in various plant species.

*Festuca elata* is a cool-season turfgrass widely used in stadiums, protective lawns, and as an urban ground cover [30]. This species is characterized by rapid growth, strong saline–alkali tolerance, wide soil adaptability, and low maintenance requirements. Under mild saline–alkali conditions, this species maintains normal growth through autonomous morphological and physiological adjustments to salt stress. However, when subjected to severe saline–alkali stress, it suffers from plasma membrane damage, diminished antioxidant capacity, significant biomass reduction, and ultimately plant death due to excessive salt stress [31,32]. Consequently, enhancing the salt tolerance of *Festuca elata* is critical for ensuring its successful establishment and growth in saline–alkali environments, which is essential not only for ecological restoration and soil improvement but also for effectively preventing further soil salinization deterioration. However, research on using exogenous melatonin to enhance salt tolerance in turfgrass remains limited; particularly, studies on the salt tolerance mechanisms of *Festuca elata* treated with exogenous MT and the optimal application concentration remain limited. Therefore, this study uses indoor pot experiments to explore these aspects and provide a reference for MT-based applications in *Festuca elata* cultivation.

## 2. Results

### 2.1. Effects of Exogenous MT on Leaf Area and Plant Height of Festuca elata Under Salt Stress

**Figure 1:**
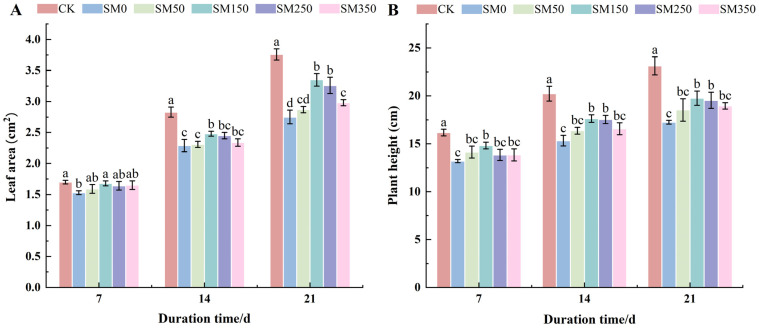
Effects of exogenous MT on leaf area and plant height of *Festuca elata* under salt stress: (**A**) changes in leaf area; (**B**) changes in plant height under different treatments. Error bars represent standard deviation (SD, *n* = 3). Different letters indicate significant differences according to Duncan’s test (*p* < 0.05).

As shown in Figure 1, compared with the control group (CK), both leaf area and plant height of SM0 decreased significantly (*p* < 0.05). Under the same stress duration, increasing the MT concentration led to an initial rise followed by a decline in both parameters, with SM150 showing the most effective response. At 21 days of stress treatment (Figure 1A), the leaf area of SM0 was 2.75 cm^2^, while that of SM150 was 3.35 cm^2^—an increase of 21.98% (*p* < 0.05). The increase observed in SM50 was not statistically significant compared with SM0 (*p* > 0.05). Similarly, Figure 1B shows that plant height increased from 17.25 cm in SM0 to 19.76 cm in SM150—a significant increase of 14.55% (*p* < 0.05). SM50 and SM350 did not show significant differences compared with SM0 (*p* > 0.05).

### 2.2. Effect of Exogenous MT on RWC of Festuca elata Under Salt Stress

**Figure 2:**
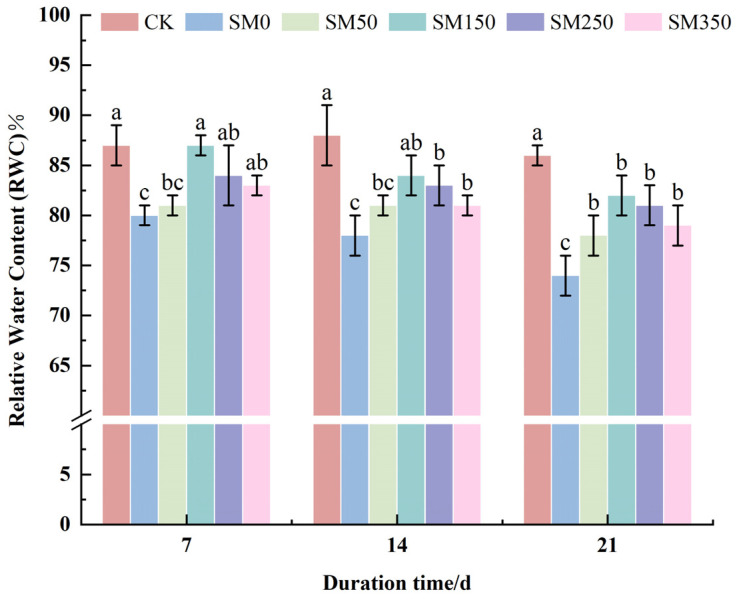
Effects of exogenous MT on relative water content (RWC) of *Festuca elata* under salt stress. Error bars represent standard deviation (SD, *n* = 3). Different letters indicate significant differences according to Duncan’s test (*p* < 0.05).

As shown in Figure 2, the relative water content (RWC) of SM0, SM50, SM150, SM250, and SM350 declined over time. However, at each stress time point, RWC generally increased initially with rising MT concentration and then decreased. After 21 days, RWC in SM0 decreased significantly by 13.55% compared with CK (*p* < 0.05), reaching a minimum of 74.01%. In contrast, SM50, SM150, SM250, and SM350 significantly improved RWC compared with SM0 (*p* < 0.05), with SM150 showing the greatest effect—raising RWC to 81.67%, a 7.66% increase over SM0 (*p* < 0.05).

### 2.3. Effects of Exogenous MT on the Content of Osmoregulatory Substances in Festuca elata Under Salt Stress

**Figure 3:**
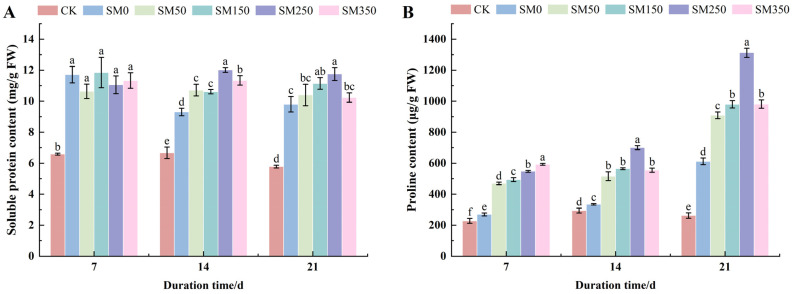
Effects of exogenous MT on osmotic substances in *Festuca elata* under salt stress: (**A**) changes in soluble protein content; (**B**) changes in proline content under different treatments. Error bars represent standard deviation (SD, *n* = 3). Different letters indicate significant differences according to Duncan’s test (*p* < 0.05).

As shown in Figure 3, compared with CK, all treatments (SM0, SM50, SM150, SM250, and SM350) significantly increased the SP and Pro contents in *Festuca elata* leaves. At 7 days, spraying different concentrations of exogenous MT under salt stress had no significant effect on SP content (*p* > 0.05), while Pro content increased significantly by 74.33–120.12% with rising MT concentration. Notably, at 14 and 21 days, SP and Pro contents showed a trend of increasing initially and then decreasing with higher MT concentrations. SM250 had the most pronounced effect, with SP content peaking at 14 days (12.02 mg/g), a significant 29.79% increase compared with SM0 (9.26 mg/g) (*p* < 0.05). Pro content reached its maximum (1312.53 μg/g) at 21 days, significantly increasing by 114.49% compared with SM0 (611.92 μg/g) (*p* < 0.05).

### 2.4. Effects of Exogenous MT on Photosynthetic Pigment Content of Festuca elata Under Salt Stress

**Figure 4:**
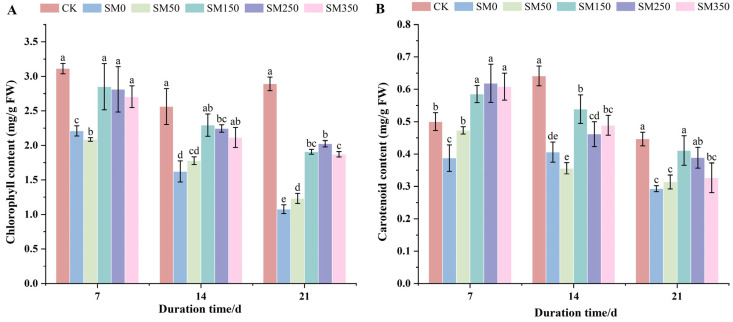
Effects of exogenous MT on photosynthetic pigments in *Festuca elata* under salt stress: (**A**) changes in chlorophyll (Chl) content; (**B**) changes in carotenoid (Car) content under different treatments. Error bars represent standard deviation (SD, *n* = 3). Different letters indicate significant differences according to Duncan’s test (*p* < 0.05).

According to Figure 4A, salt stress significantly reduced Chl content compared with CK (*p* < 0.05), and Chl content gradually declined with prolonged stress. At 7 and 14 days, SM150, SM250, and SM350 significantly increased Chl content (*p* < 0.05), with SM150 showing the greatest increases—28.95% and 41.33%, respectively, compared with SM0. At 21 days, Chl contents under SM50, SM150, SM250, and SM350 were all significantly higher than SM0 (*p* < 0.05). Chl content in SM250 reached 2.02 mg/g, an 88.15% increase compared with SM0 (1.08 mg/g).

According to Figure 4B, compared with CK, the Car content in SM0 was significantly reduced (*p* < 0.05). At 7 days, salt-stressed treatments SM50, SM150, SM250, and SM350 showed significantly increased Car content compared to SM0 (*p* < 0.05), with increases of 22.07%, 51.09%, 59.65%, and 57.01%, respectively. Under SM150, SM250, and SM350 treatment, the Car content was significantly higher than that of CK by 17.08–23.71% (*p* < 0.05). At 14 days, the Car content of SM50 showed no significant change compared with SM0 (*p* > 0.05), while SM150, SM250, and SM350 increased Car content by 9.41–50.16%, respectively, compared with SM0. At 21 days, SM50 and SM350 had no significant effect on Car content compared to SM0 (*p* > 0.05), while SM150 and SM250 significantly increased it. SM150 had the strongest effect, with Car content reaching 0.41 mg/g—an increase of 40.27% compared to SM0 (0.29 mg/g).

### 2.5. Effects of Exogenous MT on Antioxidant Enzyme Activities and MDA Content in Festuca elata Under Salt Stress

**Figure 5:**
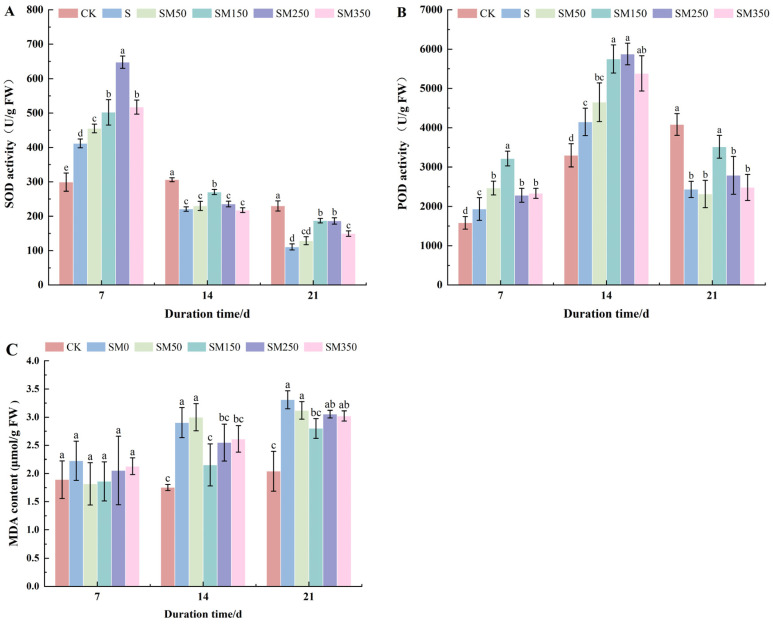
Effects of exogenous MT on antioxidant activity in *Festuca elata* under salt stress: (**A**) changes in SOD activity; (**B**) changes in POD activity; (**C**) changes in MDA content under different treatments. Error bars represent standard deviation (SD, *n* = 3). Different letters indicate significant differences according to Duncan’s test (*p* < 0.05).

According to Figure 5A, at 7 days, SM0 significantly increased the SOD activity of *Festuca elata* compared to CK (*p* < 0.05). Foliar application of MT further significantly increased SOD activity under salt stress (*p* < 0.05), with the highest value observed in SM250 (647.74 U/g/min), representing increases of 57.31% over SM0 (411.77 U/g/min) and 116.56% over CK (299.10 U/g/min). As stress duration increased, SOD activity gradually declined and became significantly lower than CK (*p* < 0.05). At 14 days, only SM150 significantly increased SOD activity compared to SM0 by 22.42% (*p* < 0.05), while SM50, SM250, and SM350 had no significant effect (*p* > 0.05). At 21 days, SM50 showed no significant effect (*p* > 0.05), but SM150, SM250, and SM350 significantly increased SOD activity (*p* < 0.05). Among these, SM150 was most effective, with SOD activity reaching 187.19 U/g/min, an increase of 68.89% over SM0 (110.837 U/g/min).

As shown in Figure 5B, the POD activity of SM0 initially increased and then decreased over time. On days 7 and 14, POD activity in SM0 was significantly higher than in CK, and spraying MT further enhanced POD activity in *Festuca elata* under salt stress (*p* <0.05). At 14 days, SOD activity in all MT treatments peaked, with SM250 showing the highest value at 5833.33 U/g/min—41.57% higher than SM0 (4150 U/g/min) and 76.77% higher than CK (3300 U/g/min). However, by day 21, SM0 POD activity dropped significantly below that of CK. Among the MT treatments, only SM150 significantly increased POD activity compared to SM0, reaching 3516.66 7 U/g/min—a 44.89% increase over SM0 (2433.333 U/g/min). SM50, SM250, and SM350 showed no significant effect on POD activity (*p* > 0.05).

Figure 5C showed that salt stress increased MDA content in *Festuca elata* leaves, with levels rising over time. At day 7, no treatment significantly affected MDA content (*p* > 0.05). However, at day 14, the MDA content in SM50 showed no significant difference compared to SM0 (*p* > 0.05), and SM150, SM250, and SM350 all demonstrated significant reductions by 9.96–25.85% (*p* < 0.05). At day 21, only SM150 significantly reduced MDA levels compared to SM0, the MDA content of SM0 reached 3.310 μmol/g—62.25% higher than CK (2.04 μmol/g). In contrast, SM150 reduced MDA to 2.80 μmol/g, a 15.41% decrease compared to SM0.

### 2.6. Principal Component Analysis of Exogenous MT on Festuca elata Under Salt Stress

**Figure 6:**
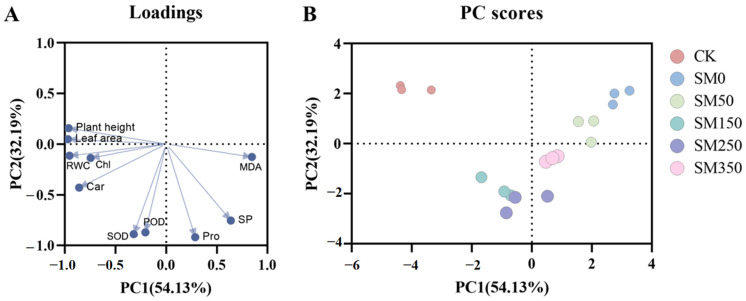
Principal component analysis of physiological indices of *Festuca elata* under NaCl stress following exogenous MT treatment: (**A**) PCA loadings; (**B**) PC scores.

Principal component analysis (PCA) was used to reduce the dimensions of 10 indicators—such as plant height, leaf area, and RWC—of *Festuca elata*. The first two principal components (PC1 and PC2) were extracted, with contribution rates of 54.13% and 32.19%, respectively, and a cumulative contribution rate of 86.32%. As shown in Figure 6A, PC1 is negatively correlated with plant height, leaf area, RWC, Chl, Car, POD, and SOD, and positively correlated with MDA, SP, and Pro. PC2 is negatively correlated with RWC, Chl, Car, POD, SOD, MDA, SP, and Pro, and positively correlated with plant height and leaf area. Plant height, leaf area, RWC, Chl, Car, and MDA contribute significantly to PC1, while SOD, POD, Pro, and SP contribute significantly to PC2. Figure 6B shows that CK and SM0 are positioned far apart, indicating a significant difference between them. CK and SM150 are positioned close together, indicating a minimal difference and suggesting that SM150 has the best mitigation effect.

### 2.7. Correlation Analysis of Exogenous MT on Festuca elata Under Salt Stress

**Figure 7:**
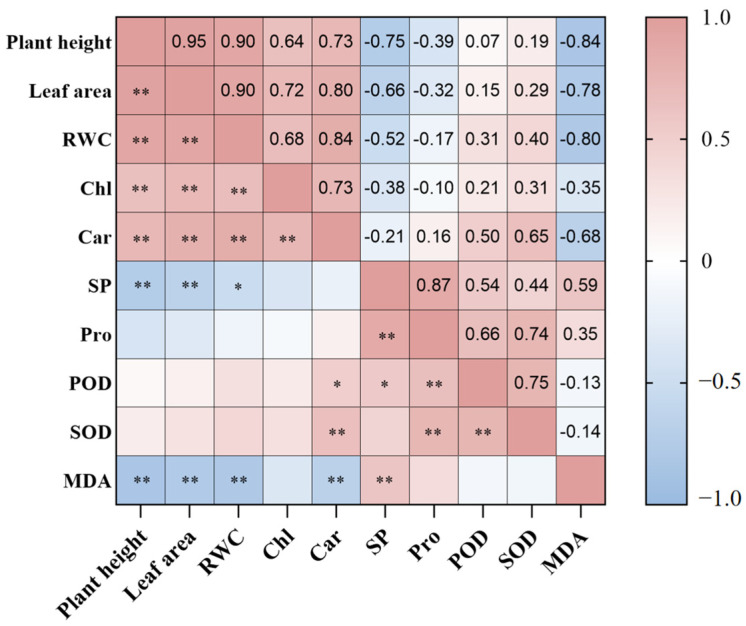
Correlation analysis of 10 morphological and physiological indices of *Festuca elata*. “*” indicates *p* < 0.05; “**” indicates *p* < 0.01.

Bivariate correlation tests were conducted on ten variables, including plant height, leaf area, RWC, Chl, Car, SP, Pro, POD, SOD, and MDA (Figure 7). Figure 7 shows that plant height and leaf area are extremely significantly positively correlated with RWC, Chl, and Car, and extremely significantly negatively correlated with SP and MDA. RWC is extremely significantly positively correlated with Chl and Car. Chl is negatively correlated with SP, Pro, and MDA, and positively correlated with POD and SOD, though none of these correlations are significant. Car is significantly positively correlated with POD, extremely significantly positively correlated with SOD, and extremely significantly negatively correlated with MDA. SP is extremely significantly positively correlated with Pro and MDA. Pro is highly significantly positively correlated with POD and SOD.

### 2.8. Comprehensive Evaluation of Membership Functions of Different Concentrations of MT in Alleviating Salt Stress of Festuca elata

The fuzzy mathematics membership function method was used to comprehensively evaluate 10 indicators of *Festuca elata* under different MT treatment concentrations. The average membership function values for six treatment groups are shown in Table 1. A higher value indicates a better effect in alleviating salt stress. The results show that, except for CK, SM150 has the highest average membership function value (0.71). The salt tolerance ranking of the six groups, from strongest to weakest, is SM150 > SM250 > CK > SM350 > SM50 > SM0. Among the five MT treatment concentrations, the order of effectiveness in alleviating salt stress is SM150 > SM250 > SM350 > SM50 > SM0.

## 3. Discussion

Growth indicators are the most direct measures reflecting plant growth status [33]. In this study, 150 μM MT (SM150) significantly alleviated the inhibitory effect of salt stress on *Festuca elata* growth (Figure 1). This aligns with previous findings [34,35]. Based on our results, it is speculated that exogenous MT enhances the antioxidant defense system and osmotic metabolism in leaves, reduces salt-induced oxidative damage, and improves plant water status, thereby mitigating growth inhibition. Similar results were reported by Ren et al. [36] and Nie et al. [37]. Xian et al. [38] further demonstrated that MT promotes plant growth under NaCl stress by increasing leaf contents of zeatin (ZT), gibberellins (GA3), and auxin (IAA), while reducing its abscisic acid (ABA) levels.

Leaf RWC reflects both the osmotic adjustment capacity and water status of leaves, making it a key indicator of plant stress resistance [39,40]. Osmotic adjustment enables plants to retain water by increasing intracellular solute concentrations, which is critical for maintaining cell osmotic pressure [41,42]. This study found that spraying exogenous MT significantly increased leaf RWC as well as SP and Pro contents (Figure 2 and Figure 3). This may result from MT-induced upregulation of genes related to Pro and SP synthesis and their associated enzyme activities, thereby increasing osmotic adjustment, antioxidant defense, and leaf water retention—helping plants survive high-salinity environments [43,44,45]. Jalili et al. [46] and Sardar et al. [47] also confirmed that exogenous MT helps plants cope with osmotic stress by increasing levels of osmotic regulators such as Pro, SP, and betaine. However, in this experiment, although salt stress (SM0) increased SP and Pro contents in *Festuca elata* leaves, RWC declined (Figure 2 and Figure 3). This may be attributed to excessive Na^+^ and Cl^−^ accumulation in the soil, which raised the soil solution’s osmotic pressure above that of root cells, hindering water absorption and leading to reduced RWC.

Chl and Car are essential for plant functions and play a key role in capturing and converting light energy [48,49]. In this study, the leaf Chl and Car contents decreased under salt stress. However, in the MT-treated group, their levels were higher than in the salt-stress group (Figure 3), consistent with previous findings [50,51]. This effect may be due to MT upregulating chlorophyll synthesis genes such as *POR*, *CAO*, and *CHLG*, and functioning as an antioxidant to scavenge excess intracellular ROS, thereby maintaining the structural and functional integrity of chloroplast membranes [52,53,54]. It is also suggested that MT may directly inhibit chlorophyll-degrading enzymes such as chlorophyllase (CLH) and pheophytinase (PPH), slowing the degradation of chlorophyll [55,56].

Salt stress induces excessive ROS production in plants, leading to oxidative stress, cellular damage, and even cell death [57]. To counter this, plants enhance antioxidant enzyme activity to reduce ROS accumulation and mitigate membrane lipid peroxidation [58]. SOD and POD are critical components of the plant antioxidant defense system. SOD catalyzes the conversion of superoxide anions into H_2_O_2_ and O_2_ [59], while POD further breaks down H_2_O_2_, protecting cells from oxidative damage [60]. MDA is a key marker of oxidative damage, indicating lipid peroxidation [61]. However, in this experiment, prolonged salt stress led to an initial increase followed by a decline in the two antioxidant enzyme activities, while MDA content continuously increased. This may be attributed to long-term high-salt-stress-damaging mitochondrial structure and function, impairing the plant’s antioxidant system [62,63]. Chen et al. [61] reported that spraying 100 μM MT increased antioxidant enzyme activities (POD and SOD) in maize leaves, reduced ROS, and enhanced salt tolerance. Our current findings are in full agreement with these observations, as MT treatment similarly elevated SOD and POD activities under salt-stress conditions (Figure 5A,B). These results corroborate previous reports by Wei et al. [64] and Khan et al. [65], further confirming the efficacy of exogenous MT in alleviating diverse abiotic stresses in plants. This effect may result from MT acting as an ROS scavenger and signaling molecule, upregulating antioxidant oxidase encoding genes, reducing ROS accumulation, and thereby preventing salt-stress-induced oxidative damage [66].

This study found that treatments with different concentrations of MT showed distinct clustering (Figure 6B), indicating that foliar application of exogenous MT can alleviate salt stress in *Festuca elata*. The order of effectiveness was SM150 > SM250 > SM350 > SM50 > SM0, consistent with the membership function analysis (Table 1). These results suggest a dose-dependent relationship between MT concentration and its alleviating effect on salt-induced plant damage. Dou et al. [67], in their study on tomato seedlings under salt-alkali stress, observed a similar trend, with the order of effectiveness being 100 μM > 150 μM > 50 μM > 200 μM > 0 μM. Similarly, Jiang et al. [29] reported that 200 μM MT had the strongest effect in mitigating salt stress, while 500 μM showed reduced efficacy—similar to the pattern observed in this experiment. Zhou et al. [68] also showed that 20 μM MT was most effective in alleviating salt-stress-induced damage in *Ginkgo biloba* seedlings, while 500 μM MT exacerbated oxidative and osmotic stress. Therefore, we speculate that low MT concentrations may fail to fully activate the antioxidant, osmotic regulation, and ion homeostasis systems due to not reaching the physiological threshold, resulting in a weaker alleviating effect. Conversely, higher MT concentrations may cause metabolic imbalance, signaling conflicts, or disrupt endogenous hormone homeostasis, thereby exerting inhibitory effects. This underlies the observed dose-dependent relationship in the effectiveness of exogenous MT in alleviating plant salt-stress damage.

MT, as a commercially available dietary supplement, offers potential cost advantages for landscaping applications due to its relatively low production cost. This study demonstrates that exogenous melatonin can significantly enhance salt tolerance in *Festuca elata* by activating antioxidant systems. However, its large-scale application requires careful evaluation, particularly due to the current lack of safety data regarding non-target organisms. Although melatonin is theoretically considered environmentally friendly, its ecological risks must be systematically assessed through experimental studies. Given the insufficient existing data, the current research remains limited to mechanistic investigations.

## 4. Materials and Methods

### 4.1. Experimental Area

The experiment was conducted in the laboratory of the Science and Technology Building at Qingdao Agricultural University. The temperature in the laboratory is 26 °C, the humidity is 35–45%, and the light intensity is approximately 800 Lux.

### 4.2. Plant Materials

The test material was the *Festuca elata* sown in flowerpots (upper radius: 30 cm; lower radius: 20 cm; height: 25 cm) in mid-April. The sowing density was 35 g/m^2^. The growth medium was a nutrient soil mix with a 1:1:1 volume ratio of vermiculite, perlite, and peat. Plants were maintained under normal conditions for 30 days.

### 4.3. Experimental Design

A randomized block design was used. Preliminary testing showed that 250 mM NaCl solution best simulated the salinity of severely saline–alkaline soils in the Shandong region, and under pot-grown conditions, *Festuca elata* treated with 250 mM NaCl for 21 days exhibited reduced growth rates, severe leaf wilting, and clear physiological stress responses. However, this salt concentration did not lead to plant death. After 30 days of normal growth, *Festuca elata* was subjected to salt stress with 250 mM NaCl. Excess salt solution was applied to maintain soil salinity, and trays were placed under pots to prevent salt loss. To avoid shock from sudden high salinity, all treatments (except the control) were gradually acclimated, starting with 50 mM NaCl and increasing by 50 mM daily until reaching 250 mM. Once the final concentration was reached, salt irrigation continued every two days. The control group was irrigated with an equal volume of clear water. Immediately after initiating salt stress, leaves were sprayed with five different concentrations of MT: 0 μM MT (SM0), 50 μM MT (SM50), 150 μM MT (SM150), 250 μM MT (SM250), and 350 μM MT (SM350). The control group (CK) was irrigated and sprayed with an equivalent volume of distilled water. Spraying occurred at 8 p.m. in the dark, using just enough solution to coat the leaf surface without dripping, approximately 150 mL was applied per pot. This treatment continued for 21 days. Samples were collected on days 7, 14, and 21 for the measurement of growth and physiological parameters. When measuring the plant height and leaf area, 10 plants were randomly selected from each pot for measurement, and the average value of each pot was calculated. The measurement of three biological replicates were repeated, and finally, the total mean of the average values of the three pots was taken. The experiment included six treatments, each with three biological replicates.

### 4.4. Determination Methods of Plant Growth and Physiological Indicators

#### 4.4.1. Leaf Area and Plant Height

The leaf area was measured using ImageJ (version 1.54g) software. Leaf blades were laid flat on a CanoScan 5600F scanner to obtain digital images, which were then imported into ImageJ to calculate blade area. Plant height was measured using a ruler, defined as the vertical distance from the base of the *Festuca elata* plant to its growth point.

#### 4.4.2. Relative Water Content

Fresh weight (FW) of the leaves was measured first. The leaves were then fully immersed in distilled water for 2 h, surface moisture was gently removed using filter paper, and the turgid weight (TW) was recorded. Finally, the leaves were dried at 80 °C until a constant weight was achieved, and the dry weight (DW) was recorded. The relative moisture content was calculated by the following formula [69].

RWC (%) = [(FW − DW)/(TW − DW)] × 100%.

#### 4.4.3. Photosynthetic Pigment Content

To determine pigment content, 0.1 g of fresh leaves was placed centrifuge tube containing 25 mL of 95% ethanol. The tubes were kept in the dark until the leaves were fully decolorized. The absorbance of the extract was measured at 665, 649, and 470 nm using a UV spectrophotometer, and Chl and Car contents were calculated [70].

#### 4.4.4. SP Content and Pro Content

SP content was determined using the Coomassie Brilliant Blue G-250 method [71], with slight modifications. Fresh leaf tissue (0.1 g) was homogenized in a mortar with quartz sand and 5 mL of phosphate buffer (pH 7.8). The homogenate was centrifuged at 15,000 rpm for 20 min at 4 °C. Subsequently, 1 mL of the supernatant was mixed with 5 mL of Coomassie Brilliant Blue solution, and the absorbance was measured at 595 nm using spectrophotometer.

Pro content was measured according to the acidic ninhydrin method [72]. Fresh leaf samples (0.5 g) were homogenized in 5 mL of 3% sulfosalicylic acid. Then, 2 mL of the extract was mixed with 2 mL of glacial acetic acid and 2 mL of ninhydrin reagent in a glass test tube. After sealing, the tubes were incubated in a boiling water bath for 40 min. The reaction mixture was extracted with toluene after cooling, and the absorbance of the extract was measured at 520 nm.

#### 4.4.5. Superoxide Dismutase Activity, Peroxidase Activity, and Malondialdehyde Content

SOD activity was determined by the nitroblue tetrazolium (NBT) method [73]. The supernatant was extracted according to the aforementioned method for determining soluble protein content. In test tubes, 1.6 mL of phosphate buffer, 0.3 mL of methionine solution, 0.3 mL of NBT solution, 0.3 mL of riboflavin solution, 0.3 mL of Ethylenediaminetetraacetic acid disodium salt (EDTA-Na_2_) solution, and 0.2 mL of supernatant were added separately. Two control tubes were prepared simultaneously, with the same reaction mixture but adding 0.2 mL of phosphate buffer instead of supernatant. One control tube was measured after light exposure as the maximum reduction tube, while the other was kept in darkness for zero adjustment during measurement. After the reaction was completed, the absorbance at 560 nm of each tube was measured using a spectrophotometer, and SOD activity was calculated. One unit of enzyme activity (U) is defined as the amount of enzyme required to inhibit the photochemical reduction in NBT by 50% under standard assay conditions.

POD activity was assayed by the guaiacol method [74]. The reaction solution was prepared by adding 0.028 mL of 3% hydrogen peroxide and 0.019 mL guaiacol to 50 mL phosphate buffer. Enzyme activity was measured by monitoring the increase in absorbance at 470 nm for 2 min after adding a 0.1 mL enzyme extract to a 3 mL reaction mixture. The values at 30 s and 90 s were used for calculation. One unit of enzyme activity (U) was defined as the amount of enzyme required to cause a change in absorbance of 0.01 per minute under the specified assay conditions.

MDA content was determined by the thiobarbituric acid (TBA) method [75]. Approximately 0.1 g of fresh leaves was weighed and placed in a mortar, to which a small amount of quartz sand and 2 mL of 10% trichloroacetic acid (TCA) were added for grinding. After grinding, the mixture was transferred to a centrifuge tube and centrifuged at 4000 r/min for 10 min. Then, 1 mL of the supernatant was taken and mixed with 1 mL of thiobarbituric acid (TBA) solution. The mixture was reacted in a boiling water bath for 15 min, quickly cooled, and centrifuged again. The supernatant was collected, and the absorbance values at 450 nm, 532 nm, and 600 nm were measured using a spectrophotometer for calculation.

### 4.5. Data Analysis

#### 4.5.1. Comprehensive Evaluation by Membership Function Method

The membership functions of each measurement index were evaluated by the following formula:

*X_i_* = (*X_i_* − *X_min_*)/(*X_max_* − *X_min_*), (1)

*X_i_* = 1 − (*X_i_* − *X_min_* )/(*X_ma_*_x_ − *X_min_*). (2)

*X_i_* is the measured value of a certain index, with *X_max_* and *X_min_* being the maximum and minimum values of this index under each treatment, respectively. The average value of the membership function was calculated for each treatment, and the stain resistance of *Festuca elata* in different treatments was measured based on the average membership function value size. The larger the value, the stronger the salt-stress resistance of *Festuca elata*. When the measured index is positively correlated with salt tolerance, the membership function Formula (1) is used, while the inverse membership function Formula (2) is used when it is negatively correlated [76].

#### 4.5.2. Data Statistics and Analysis

Data collation and organization were performed in Excel 2016. Statistical analyses were conducted using SPSS 26 (IBM Corporation, Armonk, NY, USA). Results were expressed as mean ± standard deviation (SD). The differences between treatment groups were analyzed using one-way analysis of variance (ANOVA) according to Duncan’s test (*p* < 0.05), and Pearson correlation analysis was performed. Graphs were generated using Origin 2021 and GraphPad Prism 9.

## 5. Conclusions

This study simulated salt stress to investigate the effects of exogenous MT on *Festuca elata* growth. The results showed that foliar spraying of exogenous MT reduced the salt-stress-induced growth inhibition, increased RWC and photosynthetic pigment levels, enhanced osmotic adjustment and antioxidant activity, and lowered membrane lipid peroxidation. Among the tested concentrations, 150 μM MT (SM150) had the most significant effect in improving salt tolerance in *Festuca elata*, with the order of effectiveness being 150 μM MT > 250 μM MT > 350 μM MT > 50 μM MT > 0 μM MT. These findings provide valuable insights into the application of melatonin for enhancing salt tolerance in *Festuca elata;* future studies should incorporate field trials and comprehensive long-term monitoring to establish a more robust scientific foundation for the practical application of melatonin in enhancing *Festuca elata* performance under saline conditions.

## Figures and Tables

**Table 1:** Comprehensive evaluation of alleviating *Festuca elata* under salt stress with different concentrations of MT solution.

	Plant Height	Leaf Area	RWC	Chl	Car	SP	Pro	MDA	SOD	POD	Average	Order
CK	1.00	1.00	1.00	1.00	1.00	0.00	0.00	0.11	0.28	1.00	0.64	3
SM0	0.00	0.00	0.00	0.00	0.00	0.75	0.24	0.00	0.00	0.00	0.10	6
SM50	0.24	0.12	0.28	0.05	0.11	0.81	0.63	0.23	0.22	0.18	0.29	5
SM150	0.47	0.55	0.72	0.59	0.90	0.92	0.71	1.00	0.66	0.59	0.71	1
SM250	0.37	0.45	0.55	0.59	0.76	1.00	1.00	0.60	1.00	0.28	0.66	2
SM350	0.26	0.23	0.38	0.49	0.67	0.88	0.76	0.42	0.43	0.24	0.48	4

## Data Availability

The data that support the findings of this study are available from the corresponding author upon reasonable request.
